# Stem cell–derived secretome: a novel strategy for wound healing

**DOI:** 10.3389/fphar.2026.1839998

**Published:** 2026-06-03

**Authors:** Patrícia Sousa, Ana Catarina Sousa, Alícia de Sousa Moreira, Bruna Lopes, Rui Alvites, Nuno Alves, Stefano Geuna, Ana Colette Maurício

**Affiliations:** 1 Departamento de Clínicas Veterinárias, Instituto de Ciências Biomédicas de Abel Salazar (ICBAS), Universidade do Porto (UP), Porto, Portugal; 2 LAQV, REQUIMTE, Instituto de Ciências Biomédicas de Abel Salazar, Universidade do Porto, Porto, Portugal; 3 Associate Laboratory for Animal and Veterinary Science (AL4AnimalS), Lisboa, Portugal; 4 RISE-Health, School of Medicine and Biomedical Sciences, Fernando Pessoa University, Fernando Pessoa Teaching and Culture Foundation, Gondomar, Portugal; 5 Biomedical and Health Sciences (FP-BHS), Instituto de Investigação, Inovação e Desenvolvimento Fernando Pessoa (FP-I3ID), Fundação Ensino e Cultura Fernando Pessoa, Porto, Portugal; 6 Instituto Universitário de Ciências da Saúde (CESPU), Paredes, Portugal; 7 Centre for Rapid and Sustainable Product Development, Polytechnic of Leiria, Marinha Grande, Portugal; 8 Department of Clinical and Biological Sciences, Cavalieri Ottolenghi Neuroscience Institute, University of Turin, Turin, Italy

**Keywords:** regenerative medicine, secretome, skin regeneration, stem cells, wound healing

## Abstract

Chronic non-healing wounds, including diabetic ulcers, venous leg ulcers, and pressure ulcers, represent a significant clinical burden worldwide, associated with major economic costs and impaired quality of life. Although stem cell–based therapies have shown regenerative potential, recent accumulating evidence indicates that their therapeutic effects are primarily mediated by paracrine signaling rather than direct cell engraftment. Stem cell–derived secretome, which consists of soluble bioactive factors and extracellular vesicles, has gained attention as a promising cell-free strategy for wound healing. The secretome modulates inflammation, promotes angiogenesis, supports extracellular matrix remodeling, stabilizes redox homeostasis, and enhances cell survival, proliferation, and migration, thereby creating a pro-regenerative wound microenvironment. Secretome composition is very dynamic and influenced by the stem cell source, donor characteristics, and cell culture conditions, which impacts therapeutic efficacy. Compared with live cell therapies, secretome-based approaches offer improved safety, scalability, and storage potential while avoiding risks associated with cell transplantation. This review provides an overview of the biological processes involved in wound healing, discusses the main stem cell sources used in wound healing, and summarizes current knowledge on stem cell–derived secretome and its mechanisms of action. In addition, emerging delivery strategies, therapeutic advantages, and current challenges associated with secretome-based therapies are discussed, emphasizing their potential in wound regeneration.

## Graphical Abstract

**Figure:**
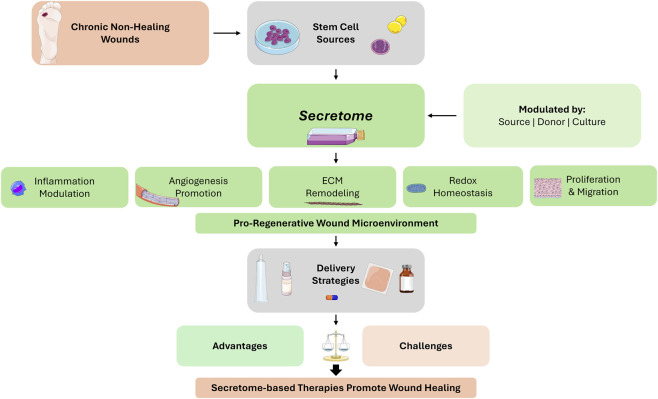

## 1 Introduction

The skin acts as a protective barrier against environmental aggressions such as microorganisms, dehydration, and ultraviolet radiation. In addition, it protects underlying organs and contributes to sensory perception, such as pain, temperature, and touch (Md Fadilah et al., 2022).

Wounds are defined as injuries that disrupt the cellular continuity, anatomical structure, or functional integrity of biological tissues. Based on the duration and progression of healing, wounds are generally classified into two main categories: acute and chronic wounds (Suhandi et al., 2023). Acute wounds follow a normal and well-organized healing process that restores the structure of the tissue. In contrast, chronic wounds do not heal properly and are characterized by ongoing inflammation, persistent infection, and even tissue necrosis (Raziyeva et al., 2021). Skin injuries are widespread and are associated with significant morbidity and mortality due to disruption of the skin’s barrier function and altered sensory perception. Damage to the skin can result from burns, cuts, abrasions, or ulcers of varying severity. Due to the skin’s essential protective role, wounds must heal efficiently to restore barrier integrity. Without appropriate treatment, breaches in the skin can compromise its protective function and expose underlying tissues to infection and further damage (Md Fadilah et al., 2022).

A wide range of therapeutic strategies are currently used in wound healing management, but the worldwide healthcare burden is still very high. This economic burden affects both human and veterinary medicine, highlighting the widespread challenges associated with wound management. In the United Kingdom, the costs are approximately £8.3 billion annually on wound management, representing 3% of its total budget. Similarly, the veterinary sector faces an increasing burden; the global animal wound care market is projected to grow to $2.6 billion by 2034, driven by advanced therapeutic needs in companion animals where complex treatments can cost up to $20,000 per patient, in the United States (Dahlgren, 2018; Carolina et al., 2023; Choi et al., 2023; Sen, 2025).

Because of their prolonged healing time, high recurrence rates, and significant economic burden, chronic wounds have become a major focus of current therapeutic strategies. Conventional wound care approaches commonly involve the use of wound dressings, topical creams and antibiotics, which serve to protect the wound from external contaminants, maintain a moist environment, and facilitate the healing process (Suhandi et al., 2023). As chronic wounds are frequently associated with concomitant metabolic diseases, such as diabetes and obesity, whose prevalence is increasing worldwide, their successful treatment and long-term management are becoming increasingly difficult (Soheilifar and Masoudi-Khoram, 2022; Yadav et al., 2025).

Despite the availability of multiple wound management strategies, many current therapies present important limitations, including high costs, increased risk of infection, frequent dressing changes, and patient discomfort. For instance, conventional wound dressings often exhibit a short residence time at the wound site, particularly in highly exuding wounds where excessive fluid accumulation can lead to dressing displacement or frequent replacement. In the case of gauze dressings, their protective function against bacterial invasion diminishes as wound exudate saturates the material, increasing the risk of infection and adherence to newly formed tissue, which may cause secondary injury during removal (Suhandi et al., 2023). Furthermore, frequent dressing changes can disrupt the healing environment, increase pain, and elevate the risk of infection, while also contributing to higher healthcare costs. In some cases, wound healing can result in scar tissue that negatively impacts patients’ lives, leading to self-esteem issues and functional limitations, even with proper care. These limitations underline the urgent need for alternative wound healing therapies that offer improved efficacy, enhanced tissue regeneration while minimizing scar formation, prolonged stability, enhanced antimicrobial protection, and greater patient safety and comfort. Such advanced approaches have the potential to address the clinical challenges associated with both acute and chronic wound care (Suhandi et al., 2023).

Cell-based therapies, particularly those involving stem cells (SCs), have shown considerable promise in wound treatment. SCs have been demonstrated to accelerate wound healing and may promote regenerative healing rather than the reparative processes that lead to fibrosis and scarring. It has been hypothesized that SCs exert some of their therapeutic effects through direct integration into regenerating tissues and differentiation into functional parenchymal cells (Ahangar et al., 2020).

Recent evidence indicates that the use of living cells in therapy can carry potential risks, like the formation of embolisms, immune reactions, or teratomas, as well as issues related to cell survival, engraftment, and tumorigenicity. In contrast, cell-derived products, such as the SCs derived secretome, bypass many of these safety concerns while retaining the regenerative and paracrine benefits of the parent cells, offering a potentially safer and more controllable approach for wound healing and tissue regeneration. Proteomic analyses of conditioned media (CM), the culture medium collected after cells have secreted these factors into it, have revealed that the cells secrete a diverse array of biologically active factors: cytokines, growth factors, messenger RNA (mRNA), extracellular vesicles, and bioactive lipids that play critical roles in skin regeneration and wound repair. Consequently, paracrine signaling, rather than direct cell replacement, is now widely regarded as the primary mechanism underlying SCs mediated healing effects (Ahangar et al., 2020).

This paradigm shift has prompted increasing interest in the therapeutic application of the SCs secretome as a cell-free alternative for wound healing, offering potential advantages such as improved safety, reduced immunogenicity, easier storage, and enhanced reproducibility compared to live cell therapies (Ahangar et al., 2020). This review highlights the regenerative potential of SCs derived-secretome in cutaneous wound healing, their origin, components and mechanism of action, and discusses the key challenges associated with its clinical translation, including standardization, delivery strategies, and regulatory considerations.

## 2 Biology of wound healing: an overview

Wounds may arise from trauma, pathological conditions, or surgical procedures and undergo a complex, multistep healing process consisting of four overlapping phases: hemostasis, inflammation, proliferation/granulation, and tissue remodeling. Proper coordination and timely progression through these phases are essential for successful healing; disruption of this sequence can result in impaired repair, chronic wounds, infections, or excessive scarring, hypertrophic scars and keloids (Lopes et al., 2021; Da Silva et al., 2025).

Chronic wounds are commonly associated with underlying conditions such as *diabetes mellitus* and other metabolic disorders, as well as with local factors including severe or deep tissue injury and inadequate wound coverage. These conditions are often accompanied by impaired keratinocyte proliferation, reduced angiogenesis, and persistent inflammation, all of which contribute to delayed healing. Immediately following injury, hemostasis occurs through platelet aggregation and fibrin clot formation via intrinsic and extrinsic coagulation pathways, temporarily sealing the wound, preventing blood loss, and providing a provisional matrix for cell migration. This event initiates the inflammatory phase, which typically lasts up to 2 days and is characterized by increased vascular permeability mediated by histamine release and the recruitment of immune cells (Lopes et al., 2021; Da Silva et al., 2025).

The inflammatory phase involves the rapid infiltration of neutrophils, followed by circulating monocytes that differentiate into macrophages and mast cells. These cells are essential for pathogen clearance, phagocytosis of debris, and regulation of subsequent healing stages through the release of cytokines and chemotactic factors. However, excessive or prolonged inflammation, particularly persistent neutrophil and macrophage activity, can lead to tissue damage through the release of reactive oxygen species and proteolytic enzymes, thereby contributing to chronic wound pathology (Lopes et al., 2021; Da Silva et al., 2025).

The proliferative phase begins approximately 3 days post-injury and may persist for up to 2 weeks. During this stage, macrophages, T lymphocytes, and platelets secrete key growth factors, including transforming growth factor-β (TGF-β), which regulates fibroblast migration, proliferation, and ECM synthesis while limiting excessive matrix degradation through the induction of tissue inhibitors of metalloproteinases. This phase is marked by angiogenesis, fibroblast migration, granulation tissue formation, and reepithelialization. Angiogenesis is stimulated by hypoxic and acidic wound conditions and is driven by growth factors, such as vascular endothelial growth factor (VEGF), basic fibroblast growth factor (bFGF), and TGF-β, resulting in the formation of a dense capillary network that supplies oxygen and nutrients to the regenerating tissue (Lopes et al., 2021; Da Silva et al., 2025).

Fibroblasts play a dominant role during proliferation by migrating into the wound bed under the influence of TGF-β and platelet-derived growth factor (PDGF), where they synthesize collagen, fibronectin, and other ECM components. The accumulation of these matrix proteins leads to granulation tissue formation, while collagen breakdown products further stimulate cell migration. In addition, epithelial cells migrate from the wound edges within hours of injury and proliferate to restore epidermal continuity, ultimately forming a new basement membrane once reepithelialization is complete (Ayavoo et al., 2021; Da Silva et al., 2025). Progression to the remodeling phase is enabled by wound contraction and epithelial closure. Remodeling may last for 6 months to 2 years and is characterized by reduced cellularity, apoptosis of fibroblasts and macrophages, cessation of angiogenesis, and gradual reorganization of the ECM. During this phase, fibroblasts differentiate into myofibroblasts, facilitating wound contraction, while initially disorganized collagen fibers are realigned, cross-linked, and strengthened, restoring up to 80% of the tissue’s original tensile strength. Dysregulation of this tightly controlled process may result in excessive scarring, hypertrophic scars, or keloid formation (Ayavoo et al., 2021; Da Silva et al., 2025).

Beyond cellular activity, successful wound healing depends on a clean wound environment and adequate local blood supply. Tissue perfusion is essential throughout all phases of healing, as it delivers oxygen, nutrients, and circulating immune cells to the wound bed, and removes metabolic waste and carbon dioxide. Ischemia, frequently encountered in diabetic foot ulcers and pressure injuries, creates a hypoxic microenvironment that impairs keratinocyte and fibroblast function, reduces angiogenesis, and prolongs the inflammatory phase, ultimately predisposing wounds to chronicity. In this context, secretome-derived pro-angiogenic factors such as VEGF, HGF, and bFGF can help restore vascular supply by stimulating endothelial cell proliferation and new capillary formation, thereby improving oxygen and nutrient delivery and creating conditions more favorable for cellular repair (Ibrahim et al., 2022; Shi et al., 2023).

In addition to circulatory compromise, local environmental factors also influence wound healing. Microbial colonization and infection disrupt all phases of repair, as microorganisms compete with host cells for nutrients, produce proteolytic enzymes and toxins that degrade the ECM and growth factors, and aid a pro-inflammatory environment dominated by neutrophil activity and elevated levels of TNF-α, IL-1β, and MMPs. Biofilm formation further complicates this, by creating a protected microbial community that resists both host immune responses and conventional antibiotic therapy. The secretome can help reduce these effects by enhancing macrophage phagocytic activity, suppressing excessive pro-inflammatory cytokine signaling, and promoting a wound microenvironment less permissive to microbial persistence. Tissue necrosis represents an additional problem, as it acts as a physical barrier to cell migration, harbors pathogenic organisms, and maintains a chronic inflammatory state that prevents progression to the proliferative phase. By modulating macrophage polarization toward the M2 phenotype and reducing proteolytic enzyme activity through downregulation of MMPs, secretome-based therapies may support the transition from a necrotic, inflammation-dominated environment to one that permits granulation tissue formation and reepithelialization. Effective wound management must therefore address perfusion, infection, and necrotic burden, in parallel with strategies aimed at restoring cellular repair processes, and the multifactorial nature of the secretome makes it particularly well-suited for this goal (Raziyeva et al., 2021; Tang et al., 2026).

Various therapeutic strategies have been developed to target specific stages of wound healing, like antibiotics to control early microbial colonization, growth factors such as epidermal growth factor (EGF), PDGF, and bFGF to enhance cell proliferation and angiogenesis, and immunomodulatory agents to regulate macrophage polarization. Despite these interventions, chronic wounds often exhibit persistent inflammation, biofilm formation, microbial colonization, and excessive exudate production. Wound exudate, rich in proteolytic enzymes, can exacerbate ECM degradation and may serve as an early indicator of systemic complications (Ayavoo et al., 2021).

Overall, effective wound healing depends on the precise temporal and spatial coordination of multiple cellular and molecular events, and therapeutic strategies aimed at restoring this balance are essential for preventing chronicity and pathological scarring (Lopes et al., 2021).

Cutaneous wound healing relies on coordinated interactions among multiple cellular and tissue components of the damaged skin, dermal and epidermal cells, the ECM, and the nervous and vascular systems. These interactions manage inflammation, angiogenesis, matrix deposition, and tissue remodeling. However, wound healing is frequently impaired in elderly individuals and patients with metabolic disorders, resulting in delayed repair and increased morbidity. Notably, fetal and adult skin exhibit distinct healing responses, fetal wounds typically heal without inflammation, contraction, or scarring, whereas adult wound healing is characterized by robust inflammation and fibrotic scar formation. These differences are largely attributed to dermal fibroblasts, the main regulators of skin regeneration, which differ between fetal and adult skin in their proliferative and migratory capacities, ability to differentiate into myofibroblasts, ECM synthesis, and responsiveness to inflammatory signals (Ayavoo et al., 2021; Md Fadilah et al., 2022).

## 3 Stem cells in regenerative medicine

### 3.1 Types of stem cells used in wound healing

Regenerative medicine is an emerging multidisciplinary field that integrates tissue engineering approaches to restore, repair or enhance the function of damaged tissues, with the goal of improving the patient’s quality of life. Within this field, stem cell–based regenerative strategies have gained significant attention due to their potential across various biomedical applications. SCs are particularly promising because of their ability to differentiate into multiple cell types and to promote tissue repair through the secretion of biologically active factors, collectively referred to as secretome (Md Fadilah et al., 2022).

SCs are undifferentiated cells with the unique capacity for self-renewal and differentiation into multiple specialized cell types, playing a fundamental role in tissue development, maintenance, and repair. These properties distinguish them from other somatic cells and also support their therapeutic potential. Under appropriate physiological or experimental conditions, SCs can proliferate and differentiate into tissue or organ-specific cells with specialized functions, and have therefore attracted considerable interest as innovative therapeutic approaches (Ayavoo et al., 2021).

SCs are primarily derived from adult tissues and embryonic sources, with accessible autologous sources, such as bone marrow, peripheral blood, umbilical cord, skin, skeletal muscle, and liver. Based on their origin and differentiation potential, SCs are broadly classified into adult stem cells (ASCs), embryonic stem cells (ESCs), and induced pluripotent stem cells (iPSCs) (Ayavoo et al., 2021).

ASCs are tissue-resident, undifferentiated cells responsible for maintaining and repairing the tissues in which they reside. Major ASC populations include hematopoietic stem cells (HSCs), mesenchymal stem cells (MSCs), neural crest stem cells (NCSCs), epithelial SCs, and skin SCs. HSCs, derived from bone marrow and peripheral blood, give rise to all blood cell lineages. MSCs are mesodermal progenitors initially identified in bone marrow by their adherence to plastic culture surfaces and capacity for multilineage differentiation. NCSCs originate from the central nervous system and differentiate into neurons and glial cells, while epithelial and skin SCs contribute to tissue renewal and play essential roles in skin regeneration and wound repair (Ayavoo et al., 2021).

ESCs are derived from the inner cell mass of *in vitro* fertilized embryos and possess pluripotent differentiation potential, enabling them to generate all cell types of the body. These cells are available only during early developmental stages and have been explored for regenerative applications involving tissues such as skin, nerve, and liver (Ayavoo et al., 2021).

iPSCs are generated by reprogramming somatic cells through the introduction of embryonic transcription factors, reverting them to a pluripotent, stem cell–like state. iPSCs combine the differentiation potential of ESCs with the ethical and immunological advantages of patient-specific cell sources (Ayavoo et al., 2021).

SCs hold significant promise in regenerative medicine due to their ability to promote tissue repair and modulate healing processes. Their applications include wound healing, bone regeneration, ocular repair, tissue engineering, and treatment of cardiovascular and neurological diseases. In wound management, SCs contribute by enhancing tissue regeneration, modulating inflammation, and promoting angiogenesis (Ayavoo et al., 2021).

### 3.2 Advantages and limitations of direct stem cell treatments

Regarding the use of stem cells, understanding their advantages and limitations is essential for their appropriate application in biomedical research and drug development. SCs play a pivotal role in regenerative medicine due to their potential for therapeutic tissue cloning, tissue regeneration, and the development of novel treatments for a wide range of diseases. In addition, they provide valuable models for studying human and animal growth, development, and disease mechanisms, and hold promise for the generation of functional tissues and organs for transplantation (Ayavoo et al., 2021).

Despite these advantages, several challenges and limitations remain. The long-term safety and biological effects of SCs–based therapies are not yet fully understood, raising concerns regarding their clinical application. Potential risks include immune rejection, genetic instability, tumorigenicity, and uncontrolled differentiation following transplantation. Moreover, while ESCs possess broad differentiation potential, they may not be suitable for treating all diseases and their use is accompanied by ethical and moral concerns, as their derivation involves the destruction of blastocysts. In addition, ESCs may form teratomas and require strict control of differentiation prior to clinical use. In contrast, ASCs are often partially specialized, which can limit their differentiation capacity, expansion potential, and therapeutic versatility. Their isolation may also be invasive, yield limited cell numbers, and be influenced by donor age or health state, affecting functional efficacy. Furthermore, SCs–based therapies face challenges related to standardization, scalability, high production costs, regulatory complexity, and difficulties in ensuring consistent quality and reproducibility. These items highlight the importance of selecting stem cell sources based on the intended application and balancing their regenerative potential against ethical, biological, technical, and safety concerns (Ayavoo et al., 2021).

## 4 Stem cell–derived secretome: concept and composition

### 4.1 Components of the secretome

The secretome refers to the full set of factors released by cells, including soluble proteins, lipids, nucleic acids, and vesicles (purified extracellular vesicles (EVs)). EVs comprise membrane-bound vesicles released into the extracellular environment, like exosomes, a small subtype of EVs of endosomal origin. It mediates intercellular communication, regulates inflammation, promotes angiogenesis, supports ECM remodeling, and enhances cell survival and migration, thereby promoting wound healing (Vizoso et al., 2017; Park et al., 2018). Due to these effects, the secretome has emerged as a key mediator of regenerative processes and a promising therapeutic strategy in regenerative medicine and wound healing.

The composition of the secretome is highly variable and influenced by multiple factors. These include the cellular source and tissue of origin, as well as donor-related characteristics such as age, sex, and health status. In addition, production conditions play a critical role, involving culture medium composition, passage number, oxygen tension, applied biochemical/mechanical stimuli, and species-specific differences. These parameters substantially affect both the qualitative and quantitative profiles of secreted bioactive components, contributing to variability in biological activity and therapeutic efficacy (Md Fadilah et al., 2022; Suhandi et al., 2023). At the molecular level, the composition of the secretome differs clearly between resting and activated states. Several immunomodulatory factors, such as prostaglandin E2 (PGE2), inducible nitric oxide synthase (iNOS), TGF-β, IL-10, hepatocyte growth factor (HGF), galectins, CCL2, CD39, CD73, TSG6, and IL-1 receptor antagonist, are expressed constitutively or under basal conditions. In contrast, exposure to inflammatory stimuli induces the expression of some regulatory molecules such as indoleamine 2,3-dioxygenase (IDO), programmed death-ligand 1 and 2 (PD-L1/PD-L2), and complement-related proteins. In addition, heme oxygenase-1 (HO-1) is predominantly expressed in resting cells and decreases upon activation. This variety of secretory profiles contributes significantly to functional heterogeneity and represents a major challenge for the standardization and clinical translation of secretome-based therapies (Kavaldzhieva et al., 2025).

**FIGURE 1:**
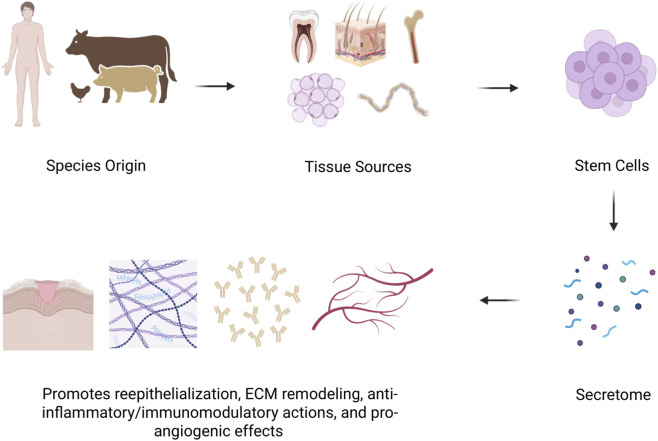
Role of stem cell–derived secretome in wound healing. Created in BioRender. Sousa, (2026) https://BioRender.com/z575gy3.

The secretome comprises a broad spectrum of soluble signaling molecules, cytokines, chemokines, growth factors, and immunomodulatory proteins, alongside EVs, such as exosomes and microvesicles. Key soluble mediators include anti and pro-regenerative cytokines such as interleukin (IL)-10, IL-6, TGF-β, and IL-1 receptor antagonist; chemokines (CCL2 and CXCL12); and growth factors such as VEGF, HGF, insulin-like growth factor 1 (IGF-1), and PDGF. These factors play central roles in cutaneous regeneration by modulating immune cell recruitment, stimulating fibroblast activation, promoting keratinocyte proliferation and migration, and supporting angiogenesis and reepithelialization (Figure 1) (Wood et al., 2014; Singampalli et al., 2020; Kim et al., 2022; Short et al., 2022).

The cytokines present within a healing wound are regulated and shift dynamically across healing phases. Pro-inflammatory cytokines including TNF-α, IL-1β, and IL-6 predominate during early inflammation and are essential for pathogen clearance and immune cell recruitment. However, their persistent elevation, as seen in chronic, infected, or ischemic wounds, actively impairs repair by sustaining MMP activity, degrading newly deposited ECM, and suppressing fibroblast and keratinocyte function. The transition to a pro-regenerative environment depends on the rise of anti-inflammatory mediators such as IL-10 and IL-4, alongside growth factors as TGF-β1, VEGF, and EGF, which collectively shift macrophage polarization toward the M2 phenotype and drive proliferative repair (Tang et al., 2021; Tang et al., 2026). Secretome is particularly promising to modulate this cytokine balance by suppressing TNF-α and IL-1β, as it can reduce the proteolytic and inflammatory burden that degrades the ECM and impairs cellular function in chronic wounds. In addition, through upregulation of IL-10 and promotion of M2 macrophage polarization, it shifts the wound environment towards a pro-regenerative state. The secretome can also directly stimulate keratinocyte proliferation and migration, fibroblast activation, and angiogenesis, thereby addressing the cellular deficits that characterize impaired wound healing. In wounds complicated by infection or necrosis, where the pro-inflammatory cytokine load is especially high and endogenous repair signals are overwhelmed, these coordinated immunomodulatory and regenerative properties of the secretome may be of particular therapeutic relevance, offering the potential to restore the sequential progression of normal wound healing phases (da Costa Pereira Cestari et al., 2026; Grogan et al., 2026).

The secretome contributes to the resolution of chronic inflammation and impaired healing. Importantly, the immunomodulatory effects of the secretome are highly context dependent. MSCs behave as dynamic sensors of their inflammatory microenvironment and adapt their secretory profile accordingly. Under low inflammatory conditions, MSC-derived signals may support immune activation and tissue surveillance, whereas exposure to high levels of pro-inflammatory cytokines such as Tumor Necrosis Factor-α (TNF-α) and Interferon-γ (IFN-γ) shifts the secretome towards a predominantly anti-inflammatory and immunosuppressive phenotype. Moreover, several mediators, for example, PGE2 and HLA-G, exert concentration-dependent effects, displaying pro-inflammatory activity at low levels and immunosuppressive activity at higher concentrations. This functional plasticity enables MSCs to fine-tune immune responses and restore homeostasis rather than inducing indiscriminate immune suppression (Kavaldzhieva et al., 2025). It can downregulate pro-inflammatory cytokines, such as TNF-α and IL-1β, promote macrophage polarization toward an anti-inflammatory M2 phenotype, and enhance keratinocyte and endothelial cell function. Additional bioactive mediators, such as, PGE2, nitric oxide, and IDO, further reinforce immunosuppressive and immunoregulatory effects by modulating T-cell proliferation, dendritic cell activity, and immune tolerance. These paracrine mechanisms are particularly relevant in skin disorders characterized by chronic inflammation, defective matrix remodeling, or insufficient angiogenesis, such as diabetic ulcers, atopic dermatitis, and psoriasis (Ashcroft et al., 2012; Deng et al., 2021; Zhou S. et al., 2022; Yang et al., 2023; Zeng et al., 2023).

Secretome components are released through both classical and non-classical secretion pathways. In the classical pathway, newly synthesized proteins bearing N-terminal signal peptides are translocated into the Endoplasmic Reticulum (ER), where they undergo post-translational modifications such as folding, glycosylation, and signal peptide cleavage. These proteins are subsequently transported to the Golgi apparatus for further processing and sorting before being secreted into the extracellular space via vesicle-mediated exocytosis. This pathway accounts for the release of many soluble cytokines and growth factors involved in tissue homeostasis and repair (Meldolesi, 2022; Wu and Krijgsveld, 2024).

By contrast, non-classical secretion pathways bypass the ER–Golgi system and enable the release of bioactive molecules either directly across the plasma membrane or through encapsulation within extracellular vesicles. EVs constitute a central component of the secretome and consist of a lipid bilayer enclosing proteins, mRNA, microRNA (miRNA), and other regulatory molecules capable of modulating gene expression and cellular behavior in recipient cells. Based on their biogenesis and size, EVs are commonly classified as microvesicles (100–1,000 nm), which originate from outward budding of the plasma membrane, or exosomes (30–100 nm), which are formed within multivesicular bodies and released upon fusion with the plasma membrane. Once released, EVs interact with target cells via ligand–receptor interactions, membrane fusion, or endocytosis, delivering their cargo and modulating signaling pathways involved in inflammation, angiogenesis, oxidative stress regulation, and tissue repair. Notably, EVs exhibit enhanced stability and prolonged circulation time compared with soluble factors, further increasing their therapeutic relevance (Hadizadeh et al., 2022; Meldolesi, 2022; Lou et al., 2024; Wu and Krijgsveld, 2024). EVs also function as active biological effectors of MSC paracrine signaling. EV cargo includes not only regulatory RNAs but also enzymes, immunomodulatory proteins, and stress-response molecules capable of directly influencing inflammation, apoptosis, and cellular metabolism in recipient cells. Through these mechanisms, MSC-derived EVs contribute to immune suppression, attenuation of oxidative stress, and enhancement of cell survival, reinforcing the concept that EVs represent a core functional unit of the secretome rather than a passive byproduct of secretion (Kavaldzhieva et al., 2025).

Importantly, the cellular source of MSCs significantly shapes secretome composition and function. Adipose Tissue–derived MSCs (AD-MSCs) typically secrete higher levels of angiogenic and anti-inflammatory mediators, whereas Bone Marrow–derived MSCs (BM-MSCs) exhibit stronger hematopoietic support and TGF-β–associated signaling profiles. Umbilical Cord–derived MSCs (UC-MSCs) often display intermediate secretory patterns, frequently associated with enhanced immunoregulatory capacity. These source-dependent differences likely contribute to variability in therapeutic outcomes and accentuate the importance of source-specific optimization when designing secretome-based strategies for wound-healing applications (Prado-Yupanqui et al., 2025; da Costa Pereira Cestari et al., 2026).

### 4.2 Paracrine mechanisms versus cell replacement

Although early regenerative medicine standards emphasized direct stem cell engraftment and differentiation as being responsible for tissue repair, recent evidence now indicates that the therapeutic benefits of stem cell–based interventions are predominantly mediated through paracrine mechanisms rather than long-term cell replacement. Several studies have demonstrated limited survival, engraftment, and differentiation of transplanted SCs in injured tissues, despite robust functional improvements, suggesting that secreted bioactive factors are the primary form of regeneration (Marques da Silva et al., 2022; Bormann et al., 2023; da Costa Pereira Cestari et al., 2026).

While the regenerative potential of the secretome is increasingly recognized, its precise molecular mechanisms remain incompletely elucidated. Current literature suggests that the biological activity of the secretome can be categorized into four interconnected mechanisms: direct stimulation of cell proliferation and migration, immunomodulation, stabilization of the redox microenvironment, and inhibition of apoptosis and cellular senescence. Among these, the promotion of cell proliferation and migration represents a primary mode of action. SCs-derived secretome has consistently demonstrated strong mitogenic and motogenic effects across multiple cell types, such as keratinocytes, fibroblasts, neurons, and Schwann cells. These effects are commonly mediated through activation of key intracellular signaling pathways such as PI3K/Akt, Focal Adhesion Kinase (FAK), and ERK1/2, which regulate cell survival, cytoskeletal dynamics, and migration. Both *in vitro* scratch assays and *in vivo* injury models have shown enhanced neurite outgrowth, angiogenesis, and tissue remodeling following secretome exposure, underscoring its role in accelerating structural regeneration independently of donor cell engraftment (Yi et al., 2020; Alvites et al., 2022; Pinho et al., 2022; Gouveia et al., 2023).

Beyond direct regenerative signaling, immunomodulation constitutes a central mechanism by which the secretome facilitates tissue repair. Secretome derived from several sources has been shown to attenuate excessive inflammatory responses by reducing leukocyte infiltration, suppressing pro-inflammatory cytokine expression, and promoting regulatory immune phenotypes. Increased expression of FOXP3 and IL-10, coupled with downregulation of NF-κB, TNF-α, and IL-6, indicates a shift toward an anti-inflammatory, pro-regenerative immune milieu. Additionally, secretome-mediated modulation of macrophage function, enhancing phagocytosis and debris clearance, has been implicated in improved outcomes following neural and musculoskeletal injury (Wood et al., 2014; Connor et al., 2019; Zhou et al., 2020).

An additional mechanism involves stabilization of the cellular redox microenvironment. Oxidative stress is a major contributor to tissue damage and impaired regeneration, particularly in ischemic, inflammatory, and degenerative conditions. SCs-derived secretome has been shown to reduce intracellular reactive oxygen species, while upregulating antioxidant enzymes such as superoxide dismutase, catalase, and glutathione peroxidase across multiple cell types and injury models. This redox-stabilizing capacity contributes to cellular protection and preserves regenerative potential in hostile microenvironments (Kahroba and Davatgaran-Taghipour, 2020; Yan et al., 2022; Chen et al., 2023). In addition to antioxidant enzymes and redox-modulating factors, the secretome, and particularly its EV fraction, contains heat shock proteins (Hsps), which play a central role in cellular stress adaptation and cytoprotection. Several Hsps, containing members of the Hsp70 family and co-chaperones such as Hop (Stip1), are consistently detected in EVs and contribute to immune modulation and protection against stress-induced damage. Among these, the small heat shock protein Hsp27 (HspB1) warrants particular attention due to its strong anti-apoptotic activity. Hsp27 functions as a molecular chaperone that prevents protein aggregation, stabilizes cytoskeletal dynamics, and enhances cellular tolerance to oxidative and inflammatory stress. Evidence suggests that MSCs not only express Hsp27 themselves, particularly under stress conditions, but may also promote their upregulation in recipient cells, thereby extending cytoprotective effects beyond the transplanted cells. These findings highlight protein-based stress response mechanisms as an additional, underappreciated layer of secretome-mediated tissue protection (Kavaldzhieva, Mladenov et al., 2025).

Finally, the stem cell secretome exerts potent anti-apoptotic and anti-senescent effects that support cell survival and long-term tissue regeneration. Exposure to secretome under hypoxic, oxidative, or inflammatory stress conditions enhances cell viability through modulation of apoptosis-related pathways, for example, increased Bcl-2/Bax ratios and activation of p38 MAPK signaling. In addition, secretome treatment has been associated with reduced expression of senescence markers such as p16, p21, p53 and β-galactosidase activity. Emerging evidence suggests that extracellular vesicle–associated non-coding RNAs, play a key role in mediating these effects by suppressing NF-κB and TNF-α–dependent inflammatory and senescence pathways (Jiang et al., 2019; Wang Y. et al., 2021; Liang et al., 2022; Zhou Z. et al., 2022).

At the molecular level, the regenerative effects of the secretome are mediated through key signaling pathways, involving HIF-1α/TGF-β1/SMAD and NF-κB, which regulate inflammation, angiogenesis, and tissue remodeling (Prado-Yupanqui et al., 2025). These findings support a shift from cell replacement strategies toward paracrine-mediated regeneration. Rather than functioning through direct tissue integration, stem cell–based therapies primarily act by reshaping the local microenvironment to favor repair. By promoting cell proliferation and migration, modulating immune responses, stabilizing oxidative stress, and inhibiting apoptosis and senescence, the secretome creates a permissive regenerative niche. However, a deeper understanding of these paracrine mechanisms is still essential for the rational design, standardization, and clinical translation of secretome-based therapies (Gwam et al., 2021). Rather than relying on durable engraftment or differentiation, SCs-based therapies exert their therapeutic effects primarily by reshaping the local microenvironment through a complex secretome composed of soluble mediators, extracellular vesicles, regulatory RNAs, and stress-response proteins. By integrating immunomodulation, redox stabilization, anti-apoptotic signaling, and promotion of cell proliferation and migration, the secretome establishes a permissive regenerative niche that enables endogenous repair processes to prevail (Kavaldzhieva et al., 2025).

## 5 Positive effects of secretome in wound healing

Although there are differences in methodology across different studies, preclinical studies demonstrate that SCs-derived secretome from diverse sources promotes wound healing across a wide range of cutaneous injury models, for example, diabetic wounds, full-thickness excisions, ulcers, inflammatory dermatitis, dermonecrosis, and ischemic skin injury. Regardless of the delivery route, secretome accelerated wound closure, enhanced reepithelialization, increased collagen deposition, stimulated fibroblast proliferation and migration, and promoted angiogenesis, often evidenced by elevated CD31 expression and increased vascular density, as demonstrated in Table 1. Mechanistically, these effects were mediated through modulation of inflammatory responses, activation of regenerative signaling pathways such as TGF-β1/SMAD, HIF-1α, and VEGF, and induction of autophagy-related processes that support epidermal regeneration (Lombardi et al., 2019; Prado-Yupanqui et al., 2025).

**TABLE 1:** Overview of *in vivo* models and therapeutic outcomes of stem cell–derived secretome in wound healing.

Cell source	Delivery method	*In vivo* model	Key outcomes	References
hUC-MSCs	Secretome in hyaluronate sponge	Mice Psoriasiform-like dermatitis	Reduced IL-17 A and TNF-α levels; Anti-inflammatory effects	Carrillo et al. (2023)
	Topical gel containing 10% secretome	Human diabetic and trophic ulcers	Reduced ulcer length, width, and area	Tan et al. (2023)
	Subcutaneous injection	Mice full-thickness wounds	Increased macrophage migration; Induced macrophage polarization toward the M2 phenotype; Enhanced wound closure	Liu et al. (2022)
	Topical	Mice full-thickness wounds	Significantly accelerated wound closure	Zhu et al. (2020)
	Pluronic gel + secretome	Rat full-thickness wounds	Improved re-epithelialization, vascularization, granulation maturation	Chin et al. (2025)
	Hyaluronic acid hydrogel sponge	Diabetic mice pressure wounds	Reduced wound area; Increased re-epithelialization	Palumbo et al. (2024)
	Subdermal injection	Rat full-thickness wounds	Increased closure rate; Reduced inflammation; Improved collagen density and organization	Mathen et al. (2021)
hAD-MSCs	Alginate sponge dressing	Mice full-thickness wounds	Accelerated regeneration; Increased re-epithelialization, angiogenesis, and granulation; Upregulated decorin, tenascin, EGFR.	Bari et al. (2020)
	Intradermal vs. Intravenous injection	Rabbit dermonecrosis	Fibroblast activation, neovascularization, re-epithelialization; IV delivery more effective	Rodrigues et al. (2024)
	Subcutaneous injection	Mice full-thickness wounds	Accelerated healing; Increased endothelial density and pericyte coverage	Silveira et al. (2022)
	Suckerin–spider silk fusion hydrogel	Diabetic mice full-thickness wounds	Faster closure; Increased endothelial proliferation; Reduced pro-inflammatory cells	Koh et al. (2023)
	Topical + dressing	Mice full-thickness wounds	Enhanced epithelial/dermal thickness; Increased angiogenesis; Reduced scarring	Park et al. (2018)
	Cell sheets or secretome injection	Mice full-thickness wounds	Cell sheets accelerated ulcer closure and dermal regeneration; Restored hair follicles and glands; Secretome improved vascularization but was less effective than cell sheets therapy	Alexandrushkina et al. (2020)
	Intradermal and transdermal delivery	Mice full-thickness wounds	Accelerated healing; Enhanced angiogenesis; No toxicity	An et al. (2021)
hBM-MSCs	Injection	Diabetic rat full-thickness wounds	Increased fibroblast migration; Improved vascularization; Enhanced granulation tissue remodeling	Saheli et al. (2020)
	Intradermal injection	Diabetic mice full-thickness wounds	Stimulated epidermal autophagy, cell proliferation, and migration; Activated HIF-1α/TGF-β1/SMAD axis; Enhanced re-epithelialization; TGF-β1 inhibition reduced healing	Shi et al. (2022)
	Injection	Diabetic rat full-thickness wounds	Faster closure; Improved biomechanics; Increased fibroblasts and vascularization	Pouriran et al. (2016)
	Alginate hydrogel patch	Mice full-thickness wounds	Significantly accelerated wound healing	Kwon et al. (2023)
Human MSCs–Endothelial Cells vs. hUC-MSCs	Secretome + Matrigel injection	Diabetic mice full-thickness wounds	hMSC-EC secretome superior for endothelial proliferation and healing	Ormazabal et al. (2022)
Bovine umbilical vein endothelial cell	Secretome cream (5%–15%)	Burned rats	Enhanced fibroblast activity, collagen deposition, re-epithelialization, hair follicle regeneration	Moghan et al. (2023)
Bone marrow derived monocytes	Alginate secretome bandage	Diabetic mice full-thickness wounds	Improved neovascularization, skin regeneration, nerve repair	Theocharidis et al. (2022)
Mouse-derived BM-MSCs and porcine-derived BM-MSCs	Magnetic microspheres + secretome	Mouse and pig full-thickness wounds	Reduced inflammation; Improved collagen deposition and angiogenesis	Jiang et al. (2025)
Rat UC-MSCs	Secretome injection	Burn rat model	Increased angiogenesis, collagen synthesis, cell proliferation; Modulated myofibroblasts	Dirja et al. (2025)
Human Natural Killer cells	Topical secretome	Mice full-thickness wounds	Enhanced epithelial, stromal, and endothelial repair via CCL3/4/5–CCR5 and pro-angiogenic pathways	Kim et al. (2026)
Mechanically stretched macrophages	Local injection (15% secretome)	Mice full-thickness wounds	Enhanced angiogenesis and dermal regeneration	Wei et al. (2025)
M2 monocytes	PU film coverage	Diabetic mice full-thickness wounds	Increased collagen deposition; No benefit for superficial closure	Boodhoo et al. (2025)
Human chorionic villus MSCs	PEGDA/SA/Col-I hydrogel + systemic secretome	Rat full-thickness wounds	Faster closure; Increased CD31^+^ vessels; Combination therapy superior to cells alone	Deng et al. (2025)
hADSC, hDPSC and Human WJ-MSC	Topical secretome	Rat full-thickness wounds	Increased epithelial viability, migration, proliferation; hDPSC-s and hWJSC-s most effective	Cases-Perera et al. (2022)
Human dermis MSCs and hASCs	Integra matrix + secretome	Mice full-thickness wounds	Secretome’s less effective than cells for closure and remodeling despite similar angiogenesis	Zomer et al. (2024)
Equine BM-MSCs	Injection	Mouse acute and MRSA-infected wounds	Accelerated closure; Enhanced granulation, vasculature, hair follicles; Reduced bacterial load in infected wounds	Rajesh et al. (2024)

The pro-healing effects of the secretome are particularly relevant in wounds where infection, necrosis, or poor vascularization have disturbed normal repair. In ischemic wounds, the angiogenic component of the secretome directly helps the vascular deficit that triggers impaired healing. By stimulating endothelial cell proliferation and new vessel formation, secretome delivery can restore tissue perfusion, improve oxygenation of the wound bed, and improve fibroblast and keratinocyte activity. This is particularly relevant in diabetic wounds, where microvascular compromise is a primary driver of chronicity (da Costa Pereira Cestari et al., 2026; Grogan et al., 2026).

In infected wound environments, secretome does not act as a direct antimicrobial agent, but exerts significant indirect effects that support infection control and resolution. By enhancing macrophage phagocytic capacity and promoting polarization toward the M2 phenotype, secretome improves the efficiency of pathogen clearance while simultaneously reducing the excessive inflammatory signaling that bacteria/biofilms perpetuate. This action helps disrupt the cycle of persistent inflammation and impaired repair that characterizes infected chronic wounds (Lyu et al., 2022; Suhandi et al., 2025).

In tissue necrosis, secretome also helps M2 macrophage activity and suppression of excessive MMP expression improving the transition from a necrotic wound environment to one of granulation tissue formation and reepithelialization. Furthermore, by delivering a coordinated array of cytokines, growth factors, and EVs that simultaneously target vascular insufficiency, immune dysregulation, and impaired cellular regeneration, secretome-based therapies address the multifactorial pathophysiology of the most clinically challenging wound types in a manner that single-factor approaches cannot (Boodhoo et al., 2025; Wong et al., 2025).

(Marques da Silva et al., 2022) evaluated the use of secretome on skin wound healing in rodent preclinical models and 25 studies met the inclusion criteria for qualitative analysis. Although substantial heterogeneity in experimental design and methodology led to a high risk of bias, the overall findings consistently demonstrated beneficial effects of secretome treatment. MSC-derived secretome accelerated wound closure, improved the quality of skin repair, reduced inflammation through decreased inflammatory cells and cytokines with enhanced M2 macrophage polarization, promoted complete re-epithelialization and epidermal organization, stimulated neovascularization via endothelial cell proliferation and increased pro-angiogenic factors, and improved scar quality through enhanced collagen types I and III expression and remodeling. Despite the need for greater methodological standardization and transparent reporting, the evidence supports MSC-derived secretome from various tissue sources as a promising regenerative therapy for skin wound treatment (Marques da Silva et al., 2022).

Suhandi et al. evaluated 20 high-quality studies involving 382 diabetic rat models. The findings demonstrated that secretome treatment significantly improved wound closure rates and reduced inflammatory cell infiltration, such as neutrophils and macrophages, indicating strong therapeutic potential for diabetic wound repair. However, substantial heterogeneity was observed, particularly in wound closure outcomes and fibroblast numbers. This variability is likely due in part to differences in secretome sources, as secretome derived from bone marrow, adipose tissue, and umbilical cord MSCs differs in their profiles of growth factors and cytokines, such as VEGF, IL-10, and EGF, which can differentially influence wound healing. Additional contributing factors may include variations in follow-up duration, cell source, administration routes, wound models, and sample sizes (Suhandi et al., 2025).

(Aghayan et al., 2025) showed that combining secretome with scaffold-based biomaterials significantly enhances preclinical burn wound healing by accelerating wound closure, increasing collagen deposition and angiogenesis, promoting growth factor expression, and modulating inflammatory responses (Aghayan et al., 2025).

Although *in vitro* studies and animal models remain valuable for mechanistic studies, human clinical studies provide the most relevant information regarding therapeutic efficacy. Accordingly, secretome-based approaches have increasingly been explored in preclinical studies as cell-free alternatives to conventional cell therapies for wound repair. Human models are widely regarded as the most informative for understanding the factors that modulate secretome activity in wound healing and for evaluating therapeutic efficacy. Supporting this, a recent clinical study reported significant improvement in wound healing in a 17-year-old patient treated with a topical gel containing Wharton’s Jelly MSCs (WJ-MSCs) derived secretome, resulting in marked lesion size reduction and complete wound closure (Md Fadilah et al., 2022).

Beyond individual case reports, multiple early-phase clinical trials are currently investigating secretome and EVs–based therapies for wound healing and dermatological disorders. These include studies evaluating AD-MSC exosomes (AD-MSC-exos), plasma-derived exosomes, and MSC-CM for wound repair (Phase 1 trials; NCT05475418, NCT02565264, NCT04134676, NCT04235296), as well as for hypertrophic scars and keloids (NCT05004779, NCT04326959). Additional trials are exploring secretome-based interventions for inflammatory and genetic skin diseases, such as psoriasis (commercial MSC-exosomes; NCT05523011) and dystrophic epidermolysis bullosa (commercial MSC-derived EVs; NCT04173650), as well as for skin aging (AD-MSC secretome; NCT05508191). These studies highlight the growing clinical interest in secretome-based strategies across a broad spectrum of cutaneous conditions (Ma et al., 2023).

In aesthetic and laser-assisted dermatology, secretome application has demonstrated additional benefits. In acne scar treatment, secretome use resulted in reduced pore volume, while post-laser application in wound models led to decreased microcrust formation, gradual erythema resolution, and improvements in transepidermal water loss. Molecular analyses further revealed increased expression of wound repair–associated genes, particularly type III procollagen, without significantly altering type I procollagen or elastin levels, suggesting a role in promoting regenerative rather than fibrotic healing (Suhandi et al., 2023).

Parallel to clinical investigation, several commercially available secretome-based products have been developed for dermatological and aesthetic applications. These products differ primarily in their cellular source and intended clinical use. Adiposecr™ (Secretosome, Iran) is derived from hAD-MSCs and is marketed for wound healing, hyperpigmentation, post-inflammatory changes, sun damage, rosacea, and skin dehydration. Carmell Secretome™ (Carmell, United States), produced from hBM-MSCs, is primarily positioned for anti-aging applications and the treatment of dark spots and skin redness. CF-FECS-DF (S.Biomedics, Republic of Korea) uses secretome from human dermal fibroblast spheroids and is designed to support repair of damaged skin. Exostem4Tech® (Falonlabs, Spain), derived from MSC secretome, is aimed at skin wound treatment. Together, these products illustrate the growing translational and commercial interest in secretome-based therapies for skin regeneration, wound healing and beauty purposes (Prado-Yupanqui et al., 2025).

Overall, these findings underline the robust pro-healing capacity of MSC-derived secretome and support their potential as versatile, cell-free therapeutic agents for skin repair and wound healing.

## 6 Types of stem cell–derived secretome used in wound healing

The production of stem cell–derived secretome is conceptually similar in human and veterinary medicine, but it differs in execution and oversight. Human secretome production is treated as a pharmaceutical-grade biologic process, with strict donor screening, highly standardized and often xeno-free culture conditions, controlled cell priming strategies, extensive quality control, and compliance with strict regulatory frameworks. In contrast, veterinary secretome production is more flexible and species-specific, frequently relying on a lighter regulatory burden that prioritizes practicality, cost, and rapid clinical application (Harman, 2013; Voga et al., 2020; de Sousa Moreira et al., 2025). Importantly, veterinary species often serve as essential *in vivo* models for evaluating safety and efficacy before translation into human patients, making cross-species validation a critical step before clinical translation to human patients. Identifying secretome-based treatments that are effective across both veterinary and human species not only accelerates translational research but also enhances the likelihood of developing robust, broadly applicable regenerative therapies (Zindle et al., 2021; Saeed and Martins-Green, 2023; Ghanbari et al., 2024).

Secretomes derived from different stem cell sources have demonstrated significant pro-regenerative effects in wound healing. Among the various sources of stem cell–derived secretomes, AD-MSCs and UC-MSCs have attracted the greatest research interest, while BM-MSCs have been less extensively explored in this context. This preference is largely attributed to the practical and ethical advantages of AD-MSCs and UC-MSCs, including non-invasive harvesting procedures, high proliferative capacity, and strong paracrine activity. UC-MSCs have emerged as a particularly promising source due to their high secretome yield, strong immunomodulatory profile, and suitability for serum-free culture conditions. The secretome derived from UC-MSCs has demonstrated pronounced angiogenic potential due to elevated levels of VEGF and HGF, making it especially suitable for chronic wound healing applications (Miranda et al., 2019; Jung et al., 2022; Li et al., 2024). BM-MSCs are typically cultured under normoxic or hypoxic conditions using low-serum media, which directly affect the secretion of angiogenic factors (Villatoro et al., 2019; Jiang et al., 2020). AD-MSC secretome has shown particular efficacy in diabetic ulcers and pressure sores, promoting granulation tissue formation and vascularization (Lombardi et al., 2019; Ma et al., 2021; Zhu and Quan, 2022).

Beyond mesenchymal stem cell–derived secretomes, other stem cell populations have also been explored in wound healing applications. Embryonic stem cell (ESC) secretome promotes angiogenesis, granulation tissue formation, and re-epithelialization, resulting in accelerated wound closure and increased tensile strength of regenerated skin. *In vitro*, it also enhances keratinocyte and fibroblast proliferation and migration. Similarly, dental pulp-derived stem cells (DPSCs) secrete bioactive mediators that stimulate angiogenesis, ECM synthesis and deposition, and fibroblast proliferation and migration, supporting effective tissue repair (Bae et al., 2019; Chen et al., 2019; Md Fadilah et al., 2022; Zhou Z. et al., 2022).

In addition, keratinocyte-derived secretome plays an important role in regulating ECM remodeling during wound healing. Keratinocytes secrete a broad range of cytokines and growth factors: ILs, TNF-α, interferons, TGF-β1, and PDGF, which modulate inflammation and matrix dynamics. Through indirect stimulation of autocrine anti-fibrogenic factors, keratinocytes help control excessive ECM deposition, thereby limiting scar formation and contracture, key factors influencing functional and aesthetic outcomes and overall patient quality of life (Sjoqvist et al., 2019; Yang et al., 2020; Md Fadilah et al., 2022).

The secretome of epidermal stem cells (EPSCs) has emerged as a promising regulator of wound healing and scar prevention. In preclinical models, it improves wound closure, reduces fibrosis and scar formation, and enhances the regeneration of skin appendages, nerves, and vasculature, while promoting a more physiological collagen organization (Duan et al., 2020; Yang et al., 2020).

These effects can be stimulated when the cells are cultured under 3D conditions or preconditioned with inflammatory stimuli, which facilitate macrophage polarization toward an anti-inflammatory M2 phenotype and reduce fibrosis. Cellular preconditioning strategies, particularly hypoxic culture, have been shown to further enhance secretome bioactivity (Miranda et al., 2019; Faruqu et al., 2021; Prado-Yupanqui et al., 2025). Exposure to low oxygen levels increases the secretion of pro-angiogenic and regenerative factors, for example, VEGF and bFGF, thereby promoting tissue repair. In parallel, advanced approaches such as genetic modification of MSCs to overexpress key regulatory molecules, such as HIF-1α and VEGF-α, have demonstrated improved regenerative outcomes (Wang J. et al., 2021; Zhang et al., 2021; Ye et al., 2023). These findings suggest that the secretome may represent a less invasive and highly effective alternative to traditional regenerative approaches.

## 7 Delivery strategies for secretome in wound healing

The therapeutic efficacy of stem cell–derived secretome largely depends on the delivery strategy used to ensure its stability, retention at the wound site, and sustained bioactivity. Several administration approaches have therefore been developed to optimize secretome delivery in wound healing applications, as summarized in Table 2.

**TABLE 2:** Secretome administration routes and their main advantages and disadvantages.

Administration route	Delivery systems	Advantages	Disadvantages
Topical	*Cream*	Localized delivery; minimal systemic exposure; easy administration; suitable for all wounds; reduced immunogenic risk	Limited penetration into deep tissues; rapid degradation; requires repeated application; stability depends on formulation; may ooze from the wound site
	*Spray formulation*	Uniform coverage; non-invasive; easy to apply, marketable, suitable for large wound areas	Limited penetration depth; rapid degradation; formulation-dependent stability
	*Hydrogel-based*	Sustained and controlled release; protects bioactive factors; mimics ECM; improves stability and efficacy; reduces dosing frequency	Complex formulation; potential batch variability; regulatory challenges; degradation rate
Injectable	*Direct Injection*	Higher local concentration; bypasses systemic clearance; improved bioavailability; suitable for deep/complex wounds	Invasive; potential injection-related complications; uneven distribution; dose control can be challenging
Injectable/Systemic	*Nanoparticle*	Improved stability; controlled release; protection from degradation; potential for targeted delivery	Technical complexity; scalability issues; regulatory problems; possible particle-related toxicity
Systemic	*Intravenous*	Allows treatment of widespread or inaccessible injuries; non-localized delivery	Rapid clearance by reticuloendothelial system; short half-life; risk of off-target effects; higher doses required
	*Oral*	High patient compliance; non-invasive; gastrointestinal lesions will be better treated	Low bioavailability; gastric degradation of proteins. No studies yet.
Surgical/Implant	*Scaffold-based*	Structural support for tissue regeneration; prolonged release; enhanced cell–matrix interactions; suitable for large or chronic wounds; more complex to apply	Manufacturing complexity; potential immune response and scaffold rejection; limited adaptability after implantation

Topical secretome treatments have become increasingly popular due to their ease of application, non-invasiveness, and patient-friendly nature, making them highly attractive for both clinical use and commercial development. These formulations allow uniform coverage of wounds, reduce the risk of irritation, and can be designed as creams, film-forming sprays or hydrogels that enhance retention, bioavailability, and penetration of bioactive factors. Additionally, topical delivery is particularly suitable for chronic wounds, burns, and inflammatory skin conditions, where localized, controlled exposure can promote reepithelialization, angiogenesis, and modulation of the inflammatory response, while minimizing systemic side effects (Palumbo et al., 2024; Widowati et al., 2024; Elgueta et al., 2025). Spray-based formulations offer several advantages over conventional topical formulations, including ease of application, reduced risk of skin irritation, sterility, and uniform coverage of the wound surface. Film-forming sprays create a thin, adherent layer on the wound, which enhances drug retention and promotes deeper penetration compared to traditional patches, thereby improving therapeutic efficacy (Li et al., 2022; Umar et al., 2022).

Among topical formulations, hydrogel-based delivery systems have emerged as one of the most promising biomaterial platforms for SCs secretome–based wound healing therapies. Hydrogels are three-dimensional polymeric networks derived from natural or synthetic materials that mimic key structural and biochemical properties of the extracellular matrix (ECM). Their high-water content, biocompatibility, and tunable mechanical properties create a favorable microenvironment for tissue regeneration while enabling controlled degradation and sustained release of encapsulated secretome components. Incorporation strategies, like pre-crosslinking mixing, post-fabrication adsorption, or simultaneous integration during polymerization, allow precise modulation of release kinetics while preserving the bioactivity of soluble factors and EVs. Preclinical studies consistently demonstrate that secretome-loaded hydrogels enhance tissue repair, attenuate inflammatory responses, and improve functional recovery, frequently outperforming direct administration of the secretome alone (Kucharzewski et al., 2019; Xu et al., 2020; Kwon et al., 2023).

Injectable delivery systems provide an effective and minimally invasive approach for administering the secretome in wound healing applications. Through local injection into or around the wound site, bioactive factors such as growth factors, cytokines, and EVs can directly modulate the wound microenvironment. This targeted delivery enhances cell migration, angiogenesis, and ECM remodeling while reducing systemic exposure. Injectable carriers, can further support sustained release of secretome components, promoting prolonged biological activity and accelerating tissue regeneration (Chocarro-Wrona et al., 2019; Umar, 2023).

In certain situations, such as extensive wounds or systemic inflammatory conditions, particularly those associated with underlying conditions, such as diabetes or vascular disease, systemic delivery of the secretome may also be considered. Through systemic administration, secretome-derived factors can exert immunomodulatory and pro-angiogenic effects that improve overall tissue perfusion and inflammatory regulation. However, systemic delivery faces challenges such as rapid clearance and limited accumulation at the wound site. Advances in delivery strategies, such as nanoparticle carriers and surface-modified formulations, are being explored to enhance circulation time, improve wound targeting, and maximize therapeutic outcomes in wound healing applications.

In addition to conventional delivery routes, nanoparticle-based systems offer significant advantages for secretome delivery in wound healing by improving stability, protection, and controlled release of therapeutic molecules. Encapsulation within nanoparticles shields secretome components from enzymatic degradation in the wound environment and enhances their retention at the injury site. Additionally, nanoparticles can be engineered to interact with specific cell types involved in healing, such as fibroblasts, keratinocytes, and endothelial cells. These systems support sustained or stimuli-responsive release, which can enhance angiogenesis, reduce inflammation, and promote reepithelialization, ultimately leading to improved wound closure and tissue repair (Daneshmandi et al., 2020; Suhandi et al., 2025; Yadav et al., 2025). Although most strategies rely on local or injectable delivery, alternative routes such as oral administration have also been proposed. Oral administration may represent an attractive and accessible route option for addressing localized oral pathologies or gastrointestinal lesions. However, it remains largely unexplored, and challenges such as rapid enzymatic degradation and low systemic absorption must be addressed before this route can be considered a viable clinical strategy (Umar, 2023).

Another advanced biomaterial-assisted strategy involves the incorporation of secretome into tissue-engineered scaffolds, that represents an advanced strategy by providing a biomimetic ECM-like architecture that supports integrin-mediated cell adhesion, cell migration, and tissue integration. These scaffolds facilitate localized and sustained presentation of key pro-angiogenic and regenerative factors such as VEGF, HGF, TGF-β, PGE2, and bFGF, which drive angiogenesis, re-epithelialization, immunomodulation, and ECM remodeling during wound healing. By combining structural support with controlled spatiotemporal release of bioactive cues, biomaterial-assisted delivery systems significantly enhance the stability, bioavailability, and therapeutic efficacy of MSC-derived secretome. Together, these approaches underscore the versatility and translational potential of biomaterial-based platforms for optimizing secretome-driven regenerative therapies (Li et al., 2024; Aghayan et al., 2025; Rattanachot et al., 2025).

## 8 Advantages of secretome-based therapy over other therapies

Several therapeutic strategies have been developed to enhance wound healing, like skin substitutes, platelet-rich plasma, and recombinant growth factors. However, the increasing global burden of chronic wounds highlights the limitations of current approaches and the need for more effective regenerative therapies. Skin substitutes containing living fibroblasts and/or keratinocytes have demonstrated clinical benefits: faster wound closure, improved re-epithelialization, and enhanced vascularization. However, their widespread clinical use remains limited by high production costs, complex storage requirements, and potential risks such as infection, immune rejection, and tumorigenicity. In addition, their application is often restricted to specialized clinical settings (Lopes et al., 2021; Carolina et al., 2023).

Similarly, recombinant growth factor therapies have shown promise in preclinical studies but largely failed to translate into effective clinical treatments due to high manufacturing costs, the need for large doses, limited efficacy in complex wound environments, and safety concerns. As a result, PDGF remains the only FDA-approved growth factor for wound healing, with restricted clinical use. In contrast to single factor approaches, the secretome delivers a coordinated and temporally regulated network of bioactive mediators that act synergistically across multiple phases of wound healing, better mimicking endogenous repair processes (Lopes et al., 2021; Carolina et al., 2023).

SCs-derived secretome therefore represents a convincing alternative by delivering a broad, physiologically balanced array of cytokines, growth factors, and chemokines that more accurately reflect the complexity of wound healing. Importantly, secretome-based therapies exhibit lower immunogenicity and an improved safety profile compared with live-cell approaches, while avoiding issues related to poor cell viability and immune activation following cell administration. Within the secretome, EVs, particularly exosomes, have emerged as critical bioactive components due to their stability, targeted intercellular communication, and ability to modulate gene expression through miRNA transfer. Additional advantages include easier storage, improved standardization, reduced ethical concerns, and strong off-the-shelf potential, alongside scalability, transportability, and ease of clinical use (Sousa et al., 2023; Zhou et al., 2023; Sousa et al., 2025).

These characteristics may be particularly valuable in chronic wounds, where dysregulated inflammation, impaired angiogenesis, and fibroblast dysfunction limit the effectiveness of conventional treatments. From a translational perspective, secretome-based products are more amenable to Good Manufacturing Practice (GMP) standardization, quality control, and batch-to-batch consistency than live-cell therapies, even though there are still some challenges associated with their production. Moreover, their therapeutic efficacy may be further enhanced through integration with advanced delivery systems such as hydrogels, wound dressings, or biomaterial scaffolds, enabling sustained release and improved local retention at the wound site. Collectively, these attributes highlight the potential of stem cell–derived secretome as an emerging therapeutic strategy for wound healing and tissue repair (Ahangar et al., 2020).

## 9 Challenges and limitations

Despite its strong therapeutic promise, the clinical use of secretome poses several critical challenges. One major limitation is inconsistency in production, as secretome composition varies substantially depending on the cellular source, donor age, health status, passage number, oxygen tension, culture medium, and conditioning strategies. These variables directly influence the profile of secreted bioactive molecules, resulting in batch-to-batch variability and reduced reproducibility across studies. Differences in harvesting, processing, concentration, and storage methods, including centrifugation, filtration, lyophilization, and cryopreservation, further complicate comparisons and affect the stability, bioavailability, and potency of secretome components. Compliance with pharmaceutical-grade manufacturing and GMP standards is therefore essential to improve consistency, safety, and reproducible efficacy (Bari et al., 2020; Calascibetta et al., 2025).

Comprehensive characterization of the secretome, its biochemical composition, biological activity, and the half-life of its components, remains challenging. Nevertheless, such characterization is essential for understanding its mechanisms of action and supporting its clinical translation. Advanced proteomic, transcriptomic, metabolomic, and genetic screening approaches are therefore required to elucidate the key signaling pathways responsible for enhanced wound healing, immunomodulation, and tissue regeneration (Chen et al., 2022).

Although secretome is generally considered safer than live-cell therapies, potential side effects must be carefully evaluated. While exosomes and EVs are less immunogenic than their parent cells, they may still elicit immune responses. Moreover, the immunosuppressive properties of secretome, beneficial in inflammatory and autoimmune conditions, may increase susceptibility to infection or tumor progression if improperly dosed. Currently, there is no consensus on optimal dosing strategies, with studies reporting doses based on total protein content, cell equivalents, or extracellular vesicle counts. This lack of standardized dosing metrics and poorly defined therapeutic windows emphasizes the need for rigorous dose–response and long-term safety studies (Wang et al., 2025).

Scalability and stability present additional challenges, as producing therapeutically effective amounts of secretome often requires substantially more MSCs than direct cell transplantation, increasing cost and the risk of phenotypic change with repeated cell passages. Protein instability and short half-lives further limit therapeutic durability. To overcome these issues, preconditioning strategies, such as hypoxia, inflammatory priming, genetic modification, small-molecule stimulation, and three-dimensional culture systems, have been employed to enhance and modify secretome composition. In particular, 3D cultures environments activate mechanotransduction and survival pathways, such as PI3K/Akt, MAPK, Wnt/β-catenin and YAP/TAZ, resulting in increased secretion of regenerative and angiogenic factors. These approaches support the development of customized, off-the-shelf secretome formulations designed for specific wound types or disease contexts (Wu and Krijgsveld, 2024).

Finally, regulatory challenges remain a major barrier to clinical implementation. The complex and variable nature of SCs complicates its classification within existing regulatory frameworks, and the absence of defined guidelines for manufacturing, quality control, potency testing, and clinical evaluation delays approval and commercialization. Addressing these challenges through standardized production protocols, robust characterization, validated storage methods, and well-designed clinical trials will be essential for advancing secretome therapies from experimental models to routine clinical practice (Ahangar et al., 2020; Prado-Yupanqui et al., 2025).

## 10 Future perspectives and emerging trends

Advances in stem cell therapies have enabled the development of engineered and customized secretome through targeted preconditioning and genetic modification strategies. By exposing SCs to hypoxia, cytokine stimulation, 3D culture systems, or gene editing approaches, the composition of the secretome can be selectively enriched with pro-regenerative, pro-angiogenic, and immunomodulatory factors. These engineered secretomes may improve therapeutic efficacy while potentially reducing some of the safety and logistical limitations associated with direct stem cell transplantation (Tran and Damaser, 2015; Su et al., 2023).

The therapeutic potential of secretome may be further enhanced through combination approaches that integrate secretome delivery with biomaterials. Bioengineered scaffolds, hydrogels, and wound dressings can improve stability, localization, and controlled release of secreted factors, addressing challenges related to protein degradation, tissue retention, and pharmacokinetics. Such combination therapies are particularly relevant in wound healing applications, where sustained and controlled delivery of bioactive molecules is critical for effective tissue repair (Tran and Damaser, 2015; Ye H. et al., 2023). The dose-response relationships and pharmacokinetics of the secretome remain poorly explored in the literature, yet understanding them is crucial for optimizing their therapeutic efficacy and safety in wound healing applications (Chouaib et al., 2023; Tang et al., 2024).

While the whole secretome provides a diverse pool of regenerative factors, exosomes are often highlighted for their role in packaging key repair signals into stable, lipid-bilayer vesicles. This encapsulation may protect bioactive molecules from degradation and facilitate targeted cellular uptake, potentially offering a more controlled delivery mechanism in the volatile environment of a wound. Although the whole secretome provides a broad spectrum of regenerative biomolecules and may be easier to produce than cell-based therapies, it can also contain pro-inflammatory cytokines or other undesired components. For this reason, the therapeutic use of isolated extracellular vesicles, particularly exosomes, is currently being investigated as a more defined and controllable regenerative strategy (Sousa et al., 2023; Ye H. et al., 2023; Zhou et al., 2023).

The inherent adaptability of secretome-based therapies also supports the development of personalized wound care strategies. By modifying secretome composition and delivery parameters according to injury type, disease state, or patient-specific inflammatory and regenerative profiles, secretome therapies can be tailored to optimize clinical outcomes. Considerations such as timing of administration, dosing, and patient selection further emphasize the potential for individualized treatment approaches (Tran and Damaser, 2015). While secretome-based therapies have demonstrated encouraging results in preclinical models, their translation into clinical practice remains in the early stages. Future clinical trials will need to focus on defining optimal formulations, delivery systems, and treatment windows, as well as establishing safety and efficacy across diverse patient populations. Although early clinical experiences with single cytokine therapies have yielded mixed results, secretome-based approaches, by leveraging the synergistic action of multiple bioactive factors, represent a promising next-generation of regenerative therapeutics. Continued progress in this field will depend on rigorous mechanistic studies, standardized production methods, and carefully designed clinical trials to fully realize the potential of engineered secretome in wound healing and regenerative medicine.

## 11 Conclusion

Although SCs-derived secretome has emerged as a highly promising strategy for enhancing wound healing and tissue regeneration, several challenges must be addressed before they can become clinically viable therapies. More robust *in vitro* and *in vivo* models are required to better characterize secretome activity, elucidate the underlying regenerative mechanisms, and facilitate the translation of experimental findings into clinically relevant outcomes. Additionally, practical considerations, like optimal timing and route of administration following injury, regulation and dosing of a complex mixture of bioactive factors, and safety concerns, must be carefully resolved prior to clinical implementation. Overall, the SCs-derived secretome represents a promising cell-free alternative to conventional stem cell–based regenerative therapies, with the potential to overcome several practical and safety limitations associated with cell transplantation. Moreover, its inherent flexibility allows for the development of specific, individualized therapies through modification of secretome composition or delivery strategies.

## Glossary

AD-MSCs: Adipose Tissue–derived MSCs
ASCs: Adult Stem Cells
bFGF: Basic Fibroblast Growth Factor
BM-MSCs: Bone Marrow–derived MSCs
CM: Conditioned Media
ECM: Extracellular Matrix
EGF: Epidermal Growth Factor
EPSCs: Epidermal Stem Cells
ER: Endoplasmic Reticulum
ESCs: Embryonic Stem Cells
EVs: Extracellular Vesicles
FAK: Focal Adhesion Kinase
GMP: Good Manufacturing Practice
hDPSC: Human Dental Pulp Stem Cells
HGF: Hepatocyte Growth Factor
hMSC-EC: Human Endothelial Cell-Differentiated Mesenchymal Stem Cells
HO-1: Heme Oxygenase-1
HSCs: Hematopoietic Stem Cells
IDO: Indoleamine 2,3-Dioxygenase
IFN- γ: Interferon γ
IGF-1: Insulin-like Growth Factor 1
IL: Interleukin
iNOS: Inducible Nitric Oxide Synthase
iPSCs: Induced Pluripotent Stem Cells
miRNA: MicroRNA
mRNA: Messenger RNA
MSCs: Mesenchymal Stem Cells
NCSCs: Neural Crest Stem Cells
PDGF: Platelet-Derived Growth Factor
PD-L1/PD-L2: Programmed Death-Ligand 1 and 2
PGE2: Prostaglandin E2
SCs: Stem Cells
TGF-β: Transforming Growth Factor-β
TNF-α: Tumor Necrosis Factor-α
UC-MSCs: Human Umbilical Cord Mesenchymal Stem Cells
VEGF: Vascular Endothelial Growth Factor
WJ-MSCs: Wharton’s Jelly Mesenchymal Stem Cells
